# The Book World of Medicine and Science

**Published:** 1904-12-31

**Authors:** 


### The Book World of Medicine and Science.

Handbook of Diseases of the Ear. 2nd Edition. By Richard Lake, F.R.C.S. Eng., Surgeon to the Royal Ear Hospital. (London: Baillière, Tindall, and Cox. 1904. Pp. 242. Price 6s. net.)

The student and practitioner will find in this book much valuable information regarding the diagnosis and management of diseases of the ear. After briefly considering some anatomical points, the writer describes the general and special methods of investigating aural cases. He then gives an account of the various diseases of the external, middle, and internal ears, devoting special chapters to intracranial complications, operations, and to the relation of aural diseases to life assurance. In examining the ear, Mr. Lake strongly advocates the more frequent use of the Eustachian catheter for diagnostic purpose, the reader being warned of the risks of traumatic emphysema and infection by septic instruments. The proper use of the auscultation tube is regarded as absolutely necessary whenever the middle ear is inflated for diagnosis or treatment. In acute mastoiditis a time-limit of 24 hours is recommended for definite signs of improvement to appear. Should there be no improvement operation becomes a matter of urgency, and the mastoid antrum should be opened without further delay. Special attention is drawn to tuberculosis of the middle ear, to Bezold's mastoiditis, and to post-influenzal mastoiditis. The existence of the last mentioned disease is apt to be overlooked as otorrhœa may be absent, and there may be no febrile symptoms. The danger of the disease is great, and acute meningitis is said to be more frequent in post-influenzal than in other forms of acute mastoiditis. The reader is particularly warned not to be deceived by a normal temperature in such cases. The chapter on intracranial complications is an important one. With the greater appreciation of the seriousness of persistent discharge from the ear, and of the recent improvements in the technique of complete post-aural operation for radical cure, fatal intracranial complications should be avoided. The relation of aural diseases to life assurance is the subject of the concluding chapter. We think the appearance of this edition will be much appreciated, representing as it does the teaching of such a well-known authority on otology.

Vegetarian and Simple Diet. By Col. A. R. Kenny-Herbert (Wyvern). (London: Swan Sonnenschein and Co. 1904. Pp. 450. Price 3s. 6d. net.)

That the Briton swears by the roast beef of old England and is somewhat prone to look with contempt on dishes of vegetable origin except as offsets to the same is not entirely matter for congratulation from a physiological standpoint. The evils of a dietary containing an excess of nitrogenous matter are now becoming more fully recognised and at the same time the merits of vegetables are more generally admitted though perhaps their use has been restricted by imperfect knowledge of their capabilities. The main difficulty lies in their proper cookery and this the volume before us will do much to remove, for it would be a mistake to regard it as interesting solely to the professed vegetarian, though with the exception of butter, milk, and eggs, foods of animal origin are excluded from its pages. It will be equally useful to the hostess intent on a new savoury, or to the more humble materfamilias with an eye to the reduction of the butcher's bill, while one may learn how to lunch and dine for 20 days at all seasons of the year on an attractive variety of vegetable dishes. The general directions for the various culinary operations are both excellent and suggestive, but the frequent use of French terms (though a full glossary is given) may prove an obstacle to the ordinary English domestic.

Year-Book of the Scientific and Learned Societies of Great Britain and Ireland. Compiled from official sources. (London: Charles Griffin and Company, Limited. Pp. 300. Price 7s. 6d.)

A feature of the present age is the rapid advance of specialism and the consequent multiplication of societies devoted to special subjects. This volume brings vividly before us the large annual output of intellectual work represented by the papers and addresses delivered before these numerous learned societies; and it provides a ready means of reference to the original contributions made each year before any of the societies on any subject. Thus, to take the medical side alone, a list is given of every paper which was read before each of the leading medical societies; for example, we find mention made of all the papers which were read, and discussions which took place at the annual meeting of the British Medical Association and at the meetings of its branches. Details, such as the place and date of the meetings, the names of the officers, the amount of the entrance fee and subscription for membership, are also given for each society. The volume is therefore a useful one for purposes of reference.

Herbert Fry's Royal Guide to the London Charities. Edited by John Lane. (London; Chatto and Windus. 1905. Price 1s. 6d.)

The present represents the 41st edition of this Annual. In it will be found a great deal of useful and accessible information on London Charities of every kind. So far as the hospitals are concerned we think it would render reference a good deal easier if they were not classed under such numerous and confusing headings. However, this is but a trifling drawback to a useful volume.

"Who's Who, 1905." (London: Adam and Charles Black. Price 7s. 6d. net.)

The increasing size of this most useful annual must soon, we think, become a source of considerable anxiety to its publishers. Possibly a little less generosity in the selection of the individuals whose names are considered to be worthy of mention, might be advisable, but still it is always difficult to discriminate as to who should or should not be mentioned; and at the moment at any rate the compilers are well on the right side. The present issue is certainly most complete and fully upholds the reputation of its predecessors.

"Who's Who Year Book, 1905." (London: Adam and Charles Black. Price 1s. net.)

The second issue of this handy little volume contains a large amount of useful information which it is often difficult to obtain in such a ready and convenient form. The book comprises various lists and particulars respecting Government officials, royal, national, learned and artistic societies, clubs, principal schools, steamship lines and their agents, and a great deal of matter of a similar kind, which is frequently required for purposes of reference and not always easily available. The book is distinctly necessary to all professional and business men and we can strongly commend it.

Willing's Press Guide, 1905. (London: Willings, Limited. 125 Strand, London, W.C. Price 1s.)

We have received the 32nd annual issue of this useful and handy little guide to the Press of the world. The information it contains is thoroughly revised and up to date, and we can with confidence say the book is one of the most, if not the most, complete and reliable work of its class.

